# Evolution of Hyperventilation-Induced Nystagmus in Acute Unilateral Vestibulopathy—Interpretative Model and Etiopathogenetic Hypotheses

**DOI:** 10.3390/audiolres14030037

**Published:** 2024-05-18

**Authors:** Francesco Frati, Alessandra D’Orazio, Valeria Gambacorta, Giacomo Ciacca, Giampietro Ricci, Mario Faralli

**Affiliations:** Department of Medicine and Surgery, Otorhinolaryngology, University of Perugia, 06123 Perugia, Italy

**Keywords:** hyperventilation, acute unilateral vestibulopathy, AUVP, vestibular neuritis, hyperventilation-induced nystagmus, HVIN

## Abstract

Hyperventilation induces metabolic changes that can elicit nystagmus (hyperventilation-induced nystagmus, HVIN) in various vestibular disorders, revealing vestibular imbalance and bringing out central or peripheral asymmetries. In acute unilateral vestibulopathy (AUVP, namely vestibular neuritis), hyperventilation can induce different patterns of nystagmus (excitatory, inhibitory, or negative), disclosing or modifying existing static vestibular asymmetries through its ability to invalidate compensation or increase peripheral excitability. In this context, we followed the evolutionary stages of HVIN in AUVP across 35 consecutive patients, with the goal of assessing alterations in the oculomotor pattern caused by hyperventilation over time. In the acute phase, the incidence of the excitatory pattern (and the strongly excitatory one, consisting of a reversal nystagmus evoked by hyperventilation) was significantly higher compared to the inhibitory pattern; then, a progressive reduction in the incidence of the excitatory pattern and a concomitant gradual increase in the incidence of the inhibitory one were observed in the follow-up period. Assuming the role of the ephaptic effect and the transient loss of vestibular compensation as opposing mechanisms, i.e., excitatory and inhibitory, respectively, the oculomotor pattern evoked by hyperventilation is the result of the interaction of these two factors. The data obtained allowed us to hypothesize an interpretative model regarding the pathogenetic aspects of responses evoked by hyperventilation and the etiologies of the disease: according to our hypotheses, the excitatory pattern implies a neuritic (viral) form of AUVP; instead, the inhibitory (and negative) one can be an expression of both the neuritic (viral) and vascular forms of the disease.

## 1. Introduction

The hyperventilation-induced nystagmus (HVIN) test is a procedure that can be easily performed during a bedside examination of dizzy patients without causing them excessive stress [1]. It is well reported that the metabolic changes induced by hyperventilation, such as hypocapnia, extracellular alkalosis, hypocalcemia, and intracellular hypoxia, can elicit nystagmus in various vestibular disorders, and that the HVIN test can be used to reveal vestibular imbalance due to its ability to bring out asymmetries both at the central and peripheral levels [1,2,3,4].

The HVIN test is unique because it can produce nystagmus even in the absence of the dynamic stimulation of the labyrinth, thus revealing unilateral vestibular disease, which is why it is very sensitive to diseases affecting the central nervous system or the vestibular nerve [2,4,5,6]. A positive test has been reported in 91.7% of cases of acoustic neuroma (vestibular schwannoma), in 75% of cases of multiple sclerosis, and in 72.7% of cerebellar diseases [6]. Acute unilateral vestibular neuritis, also known as acute unilateral vestibulopathy (AUVP), is one of the most common causes of vertigo. Despite this, to date, data about HVIN test in AUVP are limited [7]. The disease, consisting of an acute loss of unilateral vestibular function, is mainly due to two different mechanisms: a reactivation of herpes simplex virus type 1 (HSV-1) infection, which is latent in the vestibular ganglia after a primary infection, leading to an inflammatory neural process involving the vestibular nerve, or vascular damage of the vestibular receptors because of an infarction of the anterior/superior vestibular artery, which is more vulnerable than the inferior one [8,9]. A previous study observed HVIN in 77.2% of cases of acute vestibular neuritis and 37.6% of cases of compensated vestibular neuritis [6]. Both nystagmus, directed towards the affected side (excitatory pattern) and towards the healthy side (inhibitory pattern), have been described [1,6,10]. The inhibitory (or paretic) pattern seems to be due to a disruption of the central compensatory mechanisms of peripheral vestibular asymmetry, worsening the cerebellar inhibitory function [1,6,10]. On the other hand, there are several hypotheses about the underlying mechanisms of the excitatory (or irritative) one, such as neuronal hyperexcitability in only partially damaged fibers, causing a transient up-regulation of central compensatory mechanisms, the modification of membrane channel thresholds, or the transient improvement in nerve conduction along micro-demyelinated pathways (ephaptic effect) [1,6,10]. The excitatory response is less common, it can be observed almost exclusively during the acute phase, and it tends to be replaced by an inhibitory pattern during follow-up [10]. The test seems to correlate with the severity of dizziness, but a clear prognostic value has not yet emerged [10]. Moreover, in the presence or absence of modifications induced by hyperventilation to the nystagmus, it does not hold topodiagnostic significance in the differential diagnosis between the peripheral and central forms. In summary, hyperventilation can bring out or modify an existing vestibular imbalance through its ability to invalidate compensation mechanisms and/or increase peripheral excitability. Precisely for this characteristic, the test has a high sensitivity in all those vestibular pathologies that involve the auditory vestibular nerve or the central systems responsible for controlling balance [1,6]. On this basis, we wanted to follow the evolutionary stages of AUVP in a group of consecutive patients who came under our clinical observation. The aim of our study was to evaluate the changes over time in the oculomotor pattern evoked by hyperventilation in patients suffering from AUVP. The data obtained allowed us to hypothesize an interpretative model regarding the pathogenetic aspects of the responses evoked by hyperventilation and the etiologies of the disease.

## 2. Materials and Methods

**Figure 1:**
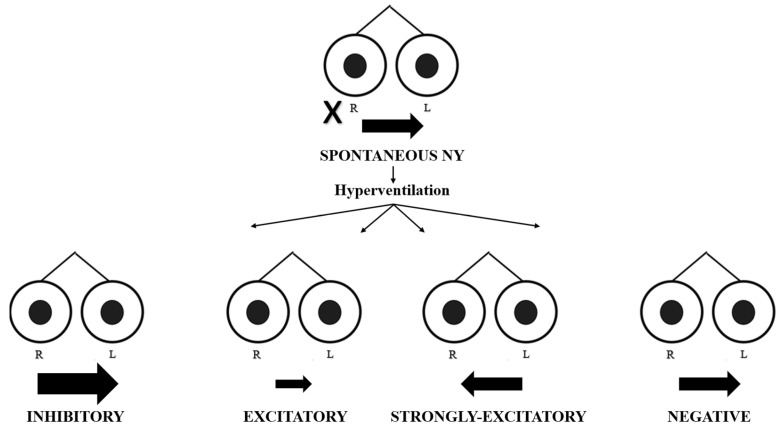
Spontaneous nystagmus and different oculomotor patterns evoked by hyperventilation. R = right eye; L = left eye. The black cross indicates the affected side. The black arrow indicates the direction and intensity of nystagmus.

**Figure 2:**
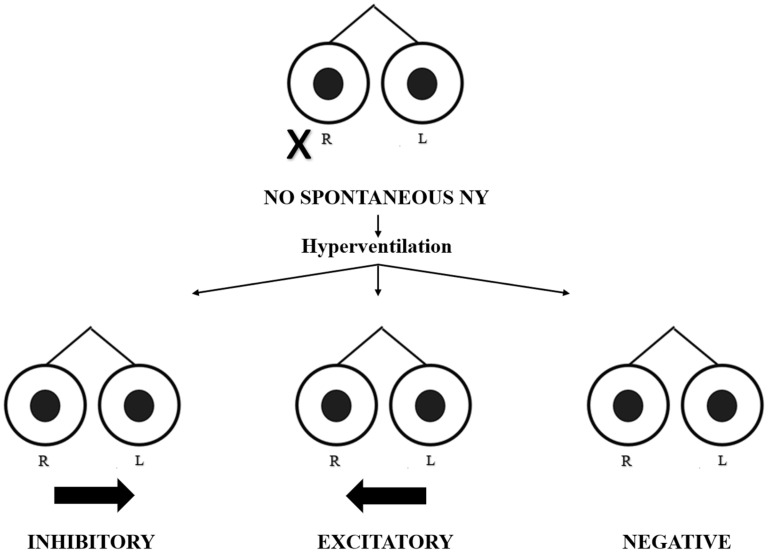
Different oculomotor patterns evoked by hyperventilation in the absence of spontaneous nystagmus. R = right eye; L = left eye. The black cross indicates the affected side. The black arrow indicates the direction and intensity of nystagmus.

We evaluated 35 patients with acute vertigo (21 M, 14 F, and mean age of 54.06 ± 14.64 years) between December 2021 and December 2022. All the patients fulfilled the criteria for AUVP according to the Bárány Society: the presence of spontaneous unidirectional nystagmus; a history of rotatory vertigo with an acute onset lasting more than 24 h and positive clinical head impulse test (HIT); and the absence of hearing loss and contralateral normal vestibular and cochlear function [7]. A contrast-enhanced MRI of the brain is always required in order to exclude a central lesion or a vestibular schwannoma, and all patients received the same medical therapy. All patients recruited into the study underwent an HVIN test. The first assessment was performed during the acute phase, within 48 h of the onset of the symptoms (mean time = 32 ± 12 h). Subsequent assessments were performed at 1 week, 1 month, and 2 months after the event in order to evaluate the patients during the sub-acute and chronic phases. The hyperventilation test was conducted with patients in a sitting position and their eyes in a primary position, asking them to take one deep breath per second for approximately 90 s; this modality has proven sufficient to lower the serum carbon dioxide (CO_2_) level enough to act on the vestibular system [6]. We recorded eye movements using video Frenzel goggles. The test is considered positive if, in the absence of spontaneous nystagmus, it evokes at least five shakes of nystagmus for a period of at least 5 s or, in the case of pre-existing spontaneous nystagmus, if its variation reaches the same value at least for the same period of time [4,6]. In terms of slow-phase velocity (SPV), HVIN is present if its SPV is of at least 5°/s. within one minute after the end of hyperventilation, with a duration of at least 5 s; if spontaneous nystagmus is already present, HVIN is present if it increases or decreases SPV of the spontaneous nystagmus of at least 5°/s, for at least 5 s. [6] Thus, in the absence of spontaneous nystagmus, the test is considered positive when hyperventilation evokes a clearly identifiable nystagmus; this eventuality could occur in the follow-up foreseen by the study due to the central compensation mechanisms that tend to attenuate the static imbalance, causing the disappearance of spontaneous nystagmus. The responses were defined as follows (Figure 1 and Figure 2):

- In the presence of spontaneous nystagmus:
  - Excitatory pattern = reduction in or reversal of nystagmus (in the case of reversal of nystagmus, we named the oculomotor pattern “strongly excitatory”);
  - Inhibitory pattern = increase in nystagmus intensity and frequency;
  - Negative pattern = no changes induced in nystagmus.
- In the absence of spontaneous nystagmus:
  - Excitatory pattern = induction of nystagmus beating towards the affected side;
  - Inhibitory pattern = induction of nystagmus beating towards the unaffected side;
  - Negative pattern = no nystagmus evoked.

We calculated the percentage incidence of the various types of oculomotor patterns evoked by the hyperventilation test during the various scheduled control periods. In the study, a series of generic vascular risk factors were considered: arterial hypertension under treatment, diabetes mellitus under treatment with oral hypoglycemic agents and/or insulin, laboratory-confirmed dyslipidemia, documented ischemic heart disease and/or cerebral vasculopathy, and smoking. Based on the presence of at least two of these risk factors, the patient was defined as “vascular-type”. The incidence of vascular type patients was calculated in the group with inhibitory and negative oculomotor patterns emerged at the 1st assessment and compared to that of the excitatory pattern group. We used the chi-squared test with Yates correction to compare the percentage values. The statistical data were considered significant at *p* values < 0.05, as per standard practice. The overall distribution of the HVIN test results among patients with and without spontaneous nystagmus during the tests performed and the relative incidence (%) were calculated. The research was conducted ethically, with all study procedures being performed in accordance with the requirements of the World Medical Association’s Declaration of Helsinki. The ethical committee approval was received with Approval No. 4056/21. We obtained a written informed consent from all patients who participated in this study.

## 3. Results

**Figure 3:**
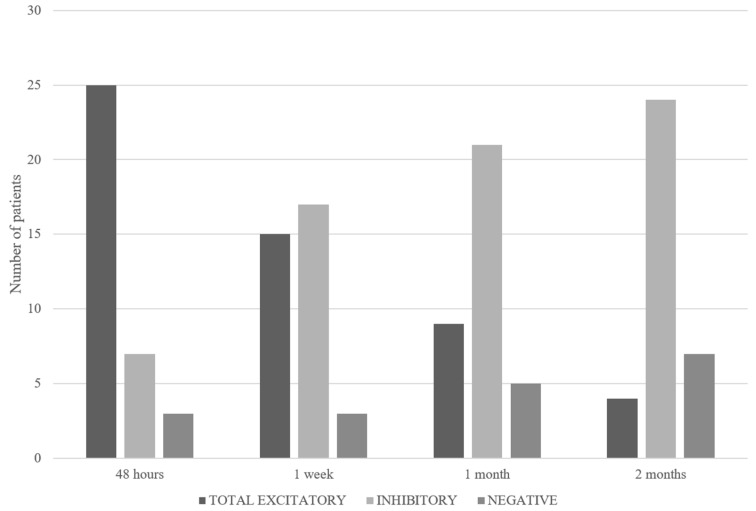
Distribution of HVIN test patterns through time. Total excitatory is inclusive of both excitatory and strongly excitatory patterns.

**Figure 4:**
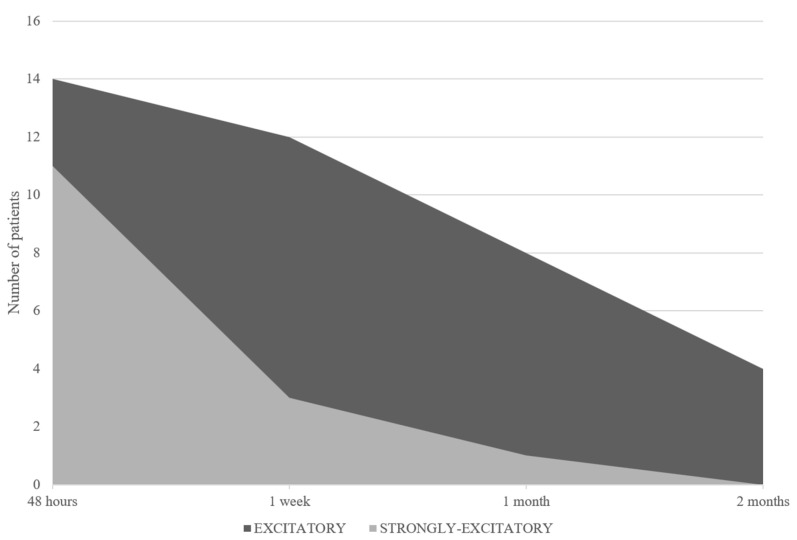
Distribution of total excitatory patterns through time: excitatory vs. strongly excitatory.

During the acute phase, all patients exhibited spontaneous nystagmus with torsional and horizontal components directed towards the healthy side, consistent with AUVP. The HVIN test was positive in 32 cases (32/35: 91.4%) (1° test). In particular, hyperventilation-induced nystagmus was excitatory in 25 patients (71.4%), inhibitory in 7 patients (20%), and in 3 (8.6%), no changes in nystagmus were induced (negative pattern) (Figure 3). The incidence (%) of the excitatory pattern at the first test was significantly greater compared to the inhibitory pattern (chi-squared statistic: 18.65; *p* = 0.000016). In the 25 patients with an excitatory pattern, a reversal of nystagmus (strongly excitatory pattern) was recorded in 11 cases (44%) (Figure 4). The “vascular-type” patients amounted to 13 (37%). Specifically, six (6/25; 24%) exhibited an excitatory oculomotor pattern, five (5/7; 71%) presented an inhibitory oculomotor pattern, and two (2/3; 66.6%) had a negative test result. Therefore, the incidence of the vascular factor was significantly higher (chi-squared statistic: 4.65; *p* = 0.03) in the group with combined inhibitory oculomotor patterns and negative test results compared to the excitatory group. After a week (2° test), the HVIN test was again positive in 32 cases (32/35: 91.4%). Of these, 15 (42.8%) showed an excitatory pattern and 17 (48.6%) an inhibitory pattern at the test. Three patients were still negative (8.6%) (Figure 3). A reversal of nystagmus (strongly excitatory pattern) affected 3 (20%) of the 15 patients with excitatory nystagmus (Figure 4). One month after the first evaluation (3° test), a positive HVIN test was recorded in 30 patients (30/35: 85.7%). In these cases, hyperventilation-induced nystagmus was excitatory in 9 patients (25.7%) and inhibitory in 21 patients (60%). In the remaining five patients (14.3%), no oculomotor response was induced by hyperventilation (negative pattern) (Figure 3). Only one (11%) of the nine patients with an excitatory pattern showed a reversal of nystagmus (strongly excitatory) (Figure 4). At the time of the last evaluation (4° test), which was performed two months from the beginning, the HVIN test was positive in 28 cases (28/35: 80%). The excitatory pattern was evoked by hyperventilation in 4 patients (11.4%) and inhibitory in 24 (68.6%). In seven patients (20%), no evoked nystagmus or no change in an existing nystagmus was present (Figure 3). None presented a strongly excitatory pattern (Figure 4). At the end of the follow-up, the reduction in the incidence of the excitatory pattern was found to be statistically significant (fourth test vs. first test; chi-squared statistic: 23.55; *p* = 0.00001). At the same time, the increase in the inhibitory pattern proved to be statistically significant (fourth test vs. first test; chi-squared statistic: 16.73; *p* = 0.000043). Spontaneous nystagmus, initially present in all 35 patients (100%), persisted in 32 (91.4%), 17 (48.6%), and 9 (25.7%) cases at the second, third, and fourth tests, respectively (Table 1). The overall distribution of HVIN test results among patients with and without spontaneous nystagmus during the tests performed and the relative incidence (%) are shown in Table 1 and Table 2.

## 4. Discussion

We observed a high percentage of positive tests during the acute phase (91.4% at 1° test), which is consistent with the data reported in other works (6). The sensitivity of the test remains relatively high even two months after the onset of symptoms (28/35; 80% at 4° test), especially in patients in whom spontaneous nystagmus persists (88.8%). The test, however, shows a good ability to reveal a functional asymmetry even in patients in whom spontaneous nystagmus has disappeared (77.7% at 3° test and 76.9% at 4° test), as shown in Table 2. The irritative pattern was less common than the inhibitory one, and its prevalence was maximal in the acute phase. Most of the patients that had an excitatory HVIN test showed a switch to an inhibitory pattern during follow-up; so, the two patterns may reflect consecutive phases. Also, the strongly excitatory pattern was an exclusive prerogative of the early stage of the disease. The chemical and metabolic changes induced by hyperventilation generate an oculomotor response through the induction of certain pathogenetic mechanisms. These are not entirely known; however, it is reasonable to hypothesize, in the case of AUVP, the intervention of at least two factors able to produce opposite effects: a transient improvement in nerve conduction due to the accumulation of extracellular ionized calcium and the transient loss of an established compensation following vestibular deafferentation (1,4,6,10). A recent study has highlighted that the finding of an inhibitory/negative HVIN test, together with other findings, should lead to the suspicion of vascular etiology [10]. The data from our study seem to confirm this hypothesis. In fact, common vascular risk factors appear to be more prevalent in those who exhibited an inhibitory or negative pattern in the earlier phases of AUVP. In this case, a receptor lesion with the preservation of the vestibular nerve can be hypothesized. On the other side, the finding of an excitatory pattern points to a neuritic form (viral) of AUVP (10). An excitatory mechanism capable of justifying the reduction in and abolition and inversion (strongly excitatory pattern) of the vestibular imbalance is represented by the induction of an ephaptic effect. The ephaptic effect consists of the exchange of information between adjacent nerve fibers (in the case of microdemyelination), resulting from the formation of an artificial synapse through their coupling. Beyond nerve fibers, the ephaptic effect can involve neurons: neurons are electrogenic and produce electric fields that can affect the electrical excitability of neighboring neurons almost instantaneously (electric field coupling) [4,11,12,13,14]. In order to propose a pathogenetic model useful for the interpretation of the semiological findings evoked by hyperventilation, for simplicity, we considered the ephaptic effect and the transient loss of vestibular compensation as opposing mechanisms, i.e., excitatory and inhibitory, respectively. These two factors can act individually or in association, and in the latter case, in a symmetrical or asymmetrical manner with the prevalence of one or the other. Therefore, the interaction of these two factors results in the oculomotor pattern that hyperventilation evokes at that particular moment.

### 4.1. Evoked Excitatory or Strongly Excitatory Oculomotor Pattern in the Presence of Spontaneous Nystagmus (Figure 5 and Table 3)

In the presence of an excitatory pattern, we must assume that an ephaptic effect generated by hyperventilation is always present, which leads to an exaltation of nerve conduction in the damaged fibers up to the induction of epileptogenic discharges. From a functional point of view, this translates into a reduction in or even an inversion (strongly-excitatory pattern) of the vestibular imbalance. Vestibular compensation may still be absent or not very pronounced, as in the very early stages of the process, or ongoing as in the developmental stages following the onset. The ephaptic effect is exerted on the affected side, while transient vestibular decompensation produces its effects mainly on the healthy side. In fact, the initial gamma-aminobutyric acid (GABA)-ergic action is expressed through an inhibitory effect mainly on the healthy side, aimed at rebalancing the discharge [15,16,17]. In any case, the inhibitory pattern induced by hyperventilation, an expression of the transient loss of vestibular compensation that tends to re-propose the initial imbalance (nystagmus), would always be of a lower entity, in terms of vestibulo-ocular reflex (VOR) gain compared to the excitatory one induced by the ephaptic effect. Based on these pathogenetic considerations, the etiology of the vestibular deficit in this case is probably viral (neuritis), as the process appears to be supported by neural damage, regardless of the presence or absence of vestibular compensation.

**Figure 5:**
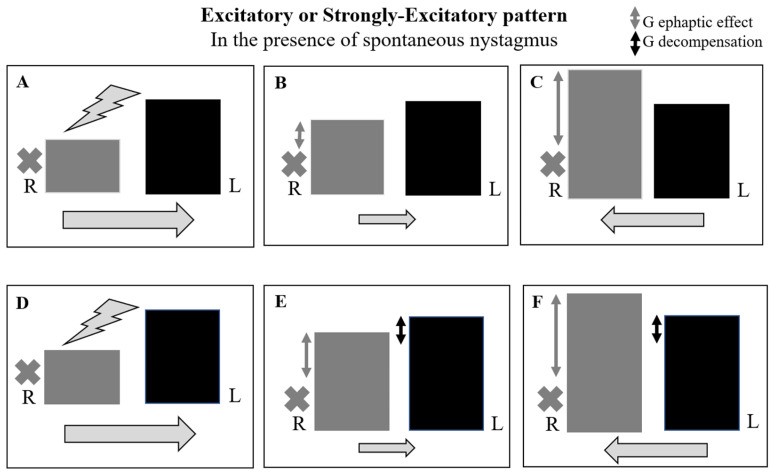
Hypothetic model for evoked excitatory or strongly excitatory oculomotor pattern in the presence of spontaneous nystagmus. In the presence of an excitatory pattern or a strongly excitatory one, we must assume that an ephaptic effect generated by hyperventilation is always present, regardless of the compensation. Compensation may be absent as in the very early stages of the disease (see the top part of the figure) or be in place as in the stages following the onset (bottom). The ephaptic effect (lightning bolt) is exerted on the affected side, while the transient loss of compensation (decompensation) produces its effects mainly on the healthy side. L: left side; R: right side. The gray cross indicates the side of the lesion. The height difference between the two columns (L vs. R) expresses the asymmetry of VOR gain between the two sides. The direction of the arrow indicates the direction of spontaneous nystagmus (to the left) or reversed nystagmus (to the right). The size of the arrow indicates the intensity of spontaneous nystagmus. Double gray arrow: ephaptic effect in terms of gain. Double black arrow: transient loss of vestibular compensation in terms of gain. (**A**–**C**): ephaptic effect present; compensation absent (acute phase). (**D**–**F**): ephaptic effect present; compensation present (sub-acute phase).

**Table 3:** Synthesis of excitatory/strongly excitatory pattern.

Ocular Pattern	Ephaptic Effect	Decompensation	Hypotesis
Excitatory or Strongly-Excitatory	Present	Absent or present but inferior to the ephaptic effect	NEURITIC

### 4.2. Evoked Excitatory Oculomotor Pattern in the Absence of Spontaneous Nystagmus (Figure 6 and Table 3)

The induction of an oculomotor response following hyperventilation can only be justified if the rebalancing of the neural discharge, which led to the disappearance of spontaneous nystagmus, was obtained through central compensation. In fact, in the case of the complete restoration of the injury (restitutio ad integrum), the lack of residual neuritic damage to the nerve capable of responding to neural stimulation, and the absence of central compensation, which is no longer needed for the restored functional state, do not explain the onset of a peri–post-hyperventilation oculomotor response as a result of induced imbalance. In this scenario, the occurrence of an excitatory pattern suggests the presence of a residual ephaptic effect that has a greater magnitude than the transient loss of compensation, which is always attained. Therefore, the presence of an ephaptic effect as an expression of neural damage points towards a viral etiology. The finding of this pattern was uncommon in our series, as it involved only two patients (patients 8 and 20).

**Figure 6:**
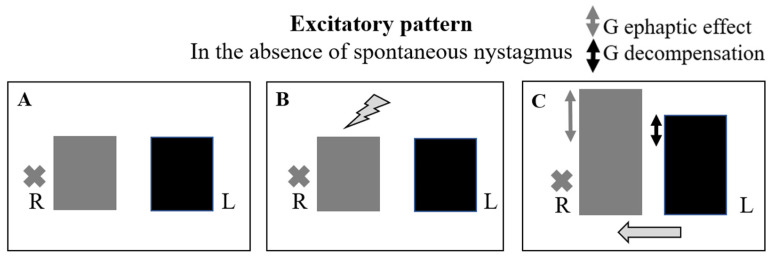
Hypothetic model for an evoked excitatory pattern in the absence of spontaneous nystagmus. Acquired rebalancing of neural discharge in the evolutionary stages of the disease. The induction of an oculomotor response following hyperventilation can only be justified if the rebalancing of the neural discharge, which leads to the disappearance of spontaneous nystagmus, is obtained through central compensation (**A**). In this case, the induction of an excitatory pattern indicates a residual ephaptic effect (**B**,**C**) of a magnitude greater than the transient loss of compensation always achieved. The viral hypothesis (neuritis) should be considered. L: left side; R: right side. The gray cross indicates the side of the lesion (R). Lightning bolt: ephaptic effect. The height difference between the two columns (L vs. R) expresses the asymmetry of VOR gain between the two sides. The direction of the arrow indicates the direction of the evoked nystagmus (to the right). Double gray arrow: ephaptic effect in terms of gain. Double black arrow: transient loss of vestibular compensation in terms of gain.

### 4.3. Evoked Inhibitory Oculomotor Pattern in the Presence of Spontaneous Nystagmus (Figure 7 and Table 4)

The induction of an inhibitory oculomotor pattern following hyperventilation always expresses the transient, partial, or total loss of vestibular compensation following the lesion, which tends to reproduce the initial nystagmus at the onset of the disease. In addition to the loss of compensation, which is certainly present, it is possible to hypothesize the coexistence of an ephaptic effect simultaneously induced by hyperventilation, the extent of which appears to be lower. The result is an increase in vestibular imbalance from a functional point of view and an increase in spontaneous nystagmus as a semiological finding. Therefore, an ephaptic effect can only be hypothesized but not proven, as it is masked, in any case, by the prevailing response of the opposite sign. Consequently, both etiologies, viral and vascular, should be considered. Twenty-one patients (60%) showed this pattern on at least one of the four scheduled tests.

**Figure 7:**
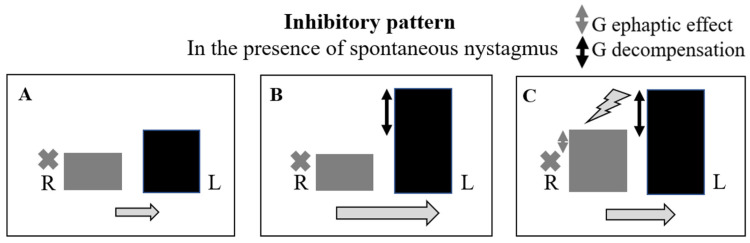
Hypothetic model for an evoked inhibitory pattern in the presence of spontaneous nystagmus. Functional situation (vestibular imbalance) before hyperventilation (**A**). An inhibitory oculomotor pattern following hyperventilation is due to a transient partial or total loss of vestibular compensation. This can occur without the simultaneous induction of an ephaptic effect, as for a receptor lesion (**B**) or it can be associated with it, as for a neural lesion. In this case, the reduction in the vestibular imbalance due to the ephaptic effect is lower than its increase secondary to the transitory vestibular decompensation (**C**). Both etiologies, viral and vascular, should be considered. L: left side; R: right side. The gray cross indicates the side of the lesion (R). Lightning bolt: ephaptic effect. The height difference between the two columns (L vs. R) expresses the asymmetry of VOR gain between the two sides. The direction of the arrow indicates the direction of the spontaneous nystagmus (to the left). Double gray arrow: ephaptic effect in terms of gain. Double black arrow: transient loss of vestibular compensation in terms of gain.

**Table 4:** Synthesis of inhibitory pattern.

Ocular Pattern	Ephaptic Effect	Decompensation	Hypotesis
Inhibitory	Present	Present and superior to the ephaptic effect	NEURITIC
	Absent	Present	VASCULAR

### 4.4. Evoked Inhibitory Oculomotor Pattern in the Absence of Spontaneous Nystagmus (Figure 8 and Table 4)

These semiological data indicate two possible conditions. The absence of an ephaptic effect or the presence of it, but to a lesser extent than the transient loss of vestibular compensation, which is always present. Also, in this case, both etiologies, viral and vascular, should be considered. This pattern proves to be the evolution of others, as it involved 19 patients during the third and fourth scheduled controls. In only one case, it was recorded on the second test and never on the first.

**Figure 8:**
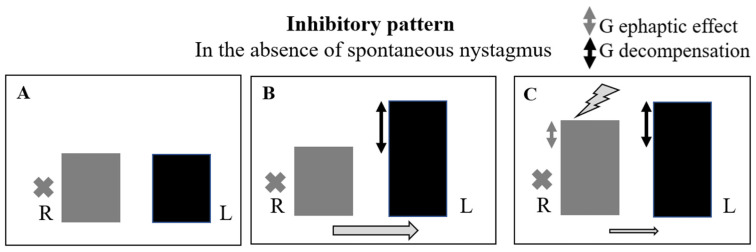
Hypothetic model for an evoked inhibitory pattern in the absence of spontaneous nystagmus. The functional state before hyperventilation is due to vestibular compensation (**A**). Transient loss of vestibular compensation without concomitant ephaptic effect (**B**). Transient loss of vestibular compensation with concomitant but smaller ephaptic effect (**C**). In this case, both etiologies, viral and vascular, should be considered. L: left side; R: right side. The gray cross indicates the side of the lesion (R). Lightning bolt: ephaptic effect. The height difference between the two columns (L vs. R) expresses the asymmetry of VOR gain between the two sides. The direction of the arrow indicates the direction of the evoked nystagmus (to the left). Double gray arrow: ephaptic effect in terms of gain. Double black arrow: transient loss of vestibular compensation in terms of gain.

### 4.5. Negative Oculomotor Pattern in the Presence of Spontaneous Nystagmus (Table 5)

A negative test leads us to consider various pathogenetic hypotheses. In the presence of spontaneous nystagmus, if an ephaptic effect due to a neuritic cause can be hypothesized, the lack of a response (negative test) can be attributed to the simultaneous transient loss of compensation of an amount equal to the ephaptic response capacity. It is a functional condition in which the two responses cancel each other out, leaving the spontaneous nystagmus unchanged. The presence of the ephaptic effect points towards the inflammatory nature of the process, although this can only be hypothesized. Secondly, spontaneous nystagmus may remain unaltered due to the lack of an ephaptic effect and the simultaneous absence of a transient loss of vestibular compensation. This is what could happen in the early stages of a vestibular deficit secondary to receptor (vascular) damage. The third circumstance involves the ineffectiveness of the test with failure to induce the ephaptic effect, and transient loss of compensation potentially present (false negative). On the basis of these speculations, it is not possible, as mentioned above, to identify an ephaptic effect; therefore, both etiologies, viral and vascular, must be considered. In our sample, only three patients show a negative pattern in the presence of spontaneous nystagmus.

**Table 5:** Synthesis of negative pattern in the presence of spontaneous nystagmus.

Ocular Pattern	Ephaptic Effect	Decompensation	Hypotesis
Negative	Present	Present and equal to the ephaptic effect	NEURITIC
	Absent	No compensation	VASCULAR
	Not evocable (ineffective test)	Not evocable (ineffective test)	NEURITIC/VASCULAR
In the presence of spontaneous nystagmus			

### 4.6. Negative Oculomotor Pattern in the Absence of Spontaneous Nystagmus (Table 6)

To define a negative pattern in the absence of spontaneous nystagmus, the documentation of a vestibular imbalance revealed by vestibular tests is required (for example, video HIT). In this situation, some anamnestic data must be considered. In the event of a negative pattern previously not negative to the hyperventilation test (positive test: excitatory or inhibitory), it is reasonable to hypothesize a restitutio ad integrum of the neural or receptor damage. The nature of the lesion may have been originally neuritic if the previous oculomotor pattern had been excitatory (patients 1, 6, and 33 in our sample) or neuritic or vascular if it had been inhibitory (patient 28 in our sample). The finding of a negative oculomotor pattern that was previously always negative in the presence of spontaneous nystagmus leads us to consider all the pathogenetic and etiological hypotheses.

**Table 6:** Synthesis of negative pattern in the absence of spontaneous nystagmus.

Ocular Pattern	Ephaptic Effect	Decompensation	Hypotesis
Negative	Not evocable (ineffective test)	Not evocable (ineffective test)	NEURITIC/ VASCULAR
Negative but previously excitatory or inhibitory	Previously present/absent (restitutio ad integrum)	Previously present/absent (restitutio ad integrum)	NEURITIC/ VASCULAR
In the absence of spontaneous nystagmus			

At present, there is no clinical marker able to resolve the differential diagnosis in the context of AUVP. In fact, an ischemic event in the territory of the anterior vestibular artery is capable of causing damage with a topographic distribution and clinical expression similar to that of superior vestibular neuritis [7,18]. Based on our hypothesis, if the finding of an excitatory (and strongly excitatory) pattern points towards a probable neural lesion and consequently a viral etiology of AUVP, the same cannot be said when an inhibitory oculomotor response to the HVIN test is evoked.

Given that the finding of an inhibitory oculomotor pattern in the early stages and not subsequent to the excitatory one places us in a position where we cannot resolve the diagnostic question, it should be considered worthy of greater attention. The possibility of ischemic vascular damage to the anterior vestibular artery, in fact, requires greater clinical surveillance as it is a possible expression of more general suffering of the posterior cerebral circulation.

## 5. Conclusions

The HVIN test shows high sensitivity in the acute phases of AUVP. It remains so even in the following stages, in the presence of spontaneous nystagmus. The test, however, proves useful in revealing a functional imbalance in many cases of the disappearance of spontaneous nystagmus. The ephaptic effect and the transient loss of vestibular compensation can be invoked as opposite pathogenetic mechanisms, i.e., excitatory and inhibitory, respectively, induced by hyperventilation. The HVIN test can be easily performed during the bedside examination and can provide some additional information about the etiology of AUVP. According to our etiopathogenetic hypothesis, based on neurophysiological observations, the excitatory oculomotor pattern points towards a viral (neural inflammatory) etiology, and the inhibitory pattern instead is unable to discern between a viral or vascular cause and, therefore, deserves more attention.

## Figures and Tables

**Table 1:** Distribution of all HVIN test results among all patients.

Patient	Nystagmus (1° Test)	Pattern (1° Test)	Nystagmus (2° Test)	Pattern (2° Test)	Nystagmus (3° Test)	Pattern (3° Test)	Nystagmus (4° Test)	Pattern (4° Test)
1	Present	Excitatory	Present	Excitatory	Present	Excitatory	Absent	Negative
2	Present	Excitatory	Present	Inhibitory	Absent	Inhibitory	Absent	Inhibitory
3	Present	Inhibitory	Present	Inhibitory	Present	Inhibitory	Absent	Inhibitory
4	Present	Strongly-Excitatory	Present	Strongly-Excitatory	Present	Strongly-Excitatory	Present	Excitatory
5	Present	Inhibitory	Present	Inhibitory	Absent	Inhibitory	Absent	Inhibitory
6	Present	Excitatory	Present	Inhibitory	Absent	Negative	Absent	Negative
7	Present	Strongly-Excitatory	Present	Excitatory	Absent	Inhibitory	Absent	Inhibitory
8	Present	Strongly-Excitatory	Present	Excitatory	Absent	Excitatory	Absent	Inhibitory
9	Present	Excitatory	Present	Inhibitory	Present	Inhibitory	Absent	Inhibitory
10	Present	Negative	Absent	Negative	Absent	Negative	Absent	Negative
11	Present	Inhibitory	Present	Inhibitory	Present	Inhibitory	Present	Inhibitory
12	Present	Excitatory	Present	Excitatory	Present	Inhibitory	Absent	Inhibitory
13	Present	Strongly-Excitatory	Present	Excitatory	Absent	Inhibitory	Absent	Inhibitory
14	Present	Strongly-Excitatory	Present	Excitatory	Present	Excitatory	Absent	Inhibitory
15	Present	Negative	Present	Negative	Present	Negative	Present	Negative
16	Present	Excitatory	Present	Inhibitory	Absent	Inhibitory	Absent	Inhibitory
17	Present	Strongly-Excitatory	Present	Excitatory	Present	Inhibitory	Absent	Inhibitory
18	Present	Strongly-Excitatory	Present	Excitatory	Present	Excitatory	Present	Inhibitory
19	Present	Inhibitory	Present	Inhibitory	Absent	Inhibitory	Absent	Inhibitory
20	Present	Strongly-Excitatory	Present	Excitatory	Absent	Excitatory	Absent	Excitatory
21	Present	Excitatory	Present	Inhibitory	Present	Inhibitory	Present	Inhibitory
22	Present	Inhibitory	Present	Inhibitory	Absent	Inhibitory	Absent	Inhibitory
23	Present	Excitatory	Present	Inhibitory	Present	Inhibitory	Present	Inhibitory
24	Present	Inhibitory	Absent	Inhibitory	Absent	Inhibitory	Absent	Inhibitory
25	Present	Strongly-Excitatory	Present	Excitatory	Present	Excitatory	Absent	Inhibitory
26	Present	Excitatory	Present	Inhibitory	Absent	Inhibitory	Absent	Inhibitory
27	Present	Excitatory	Present	Excitatory	Present	Inhibitory	Present	Inhibitory
28	Present	Inhibitory	Present	Inhibitory	Absent	Negative	Absent	Negative
29	Present	Strongly-Excitatory	Present	Strongly-Excitatory	Present	Excitatory	Present	Excitatory
30	Present	Excitatory	Present	Inhibitory	Absent	Inhibitory	Absent	Inhibitory
31	Present	Excitatory	Present	Inhibitory	Present	Inhibitory	Absent	Inhibitory
32	Present	Negative	Absent	Negative	Absent	Negative	Absent	Negative
33	Present	Excitatory	Present	Inhibitory	Absent	Inhibitory	Absent	Negative
34	Present	Strongly-Excitatory	Present	Strongly-Excitatory	Present	Excitatory	Present	Excitatory
35	Present	Excitatory	Present	Excitatory	Absent	Inhibitory	Absent	Inhibitory

**Table 2:** Incidence of HVIN test results among patients with and without spontaneous nystagmus.

Test	Spontaneous Nystagmus	No Sspontaneous nNystagmus	Positive HVIN Test	Negative HVIN Test	Incidence % Positive HVIN Test/ Spontaneous Nystagmus	Incidence % Positive HVIN Test/ No Spontaneous Nystagmus
1st	35	0	32	3	91.4%	0%
2nd	32	3	32	3	96.8%	33.3%
3rd	17	18	30	5	94.1%	77.7%
4th	9	26	28	7	88.8%	76.9%

## Data Availability

Data are contained within the article.
